# 970. The Time for Action is Now: The Impact of Timing with Infectious Diseases Consultation for *Staphylococcus aureus* Bacteremia

**DOI:** 10.1093/ofid/ofac492.812

**Published:** 2022-12-15

**Authors:** J Chase Cole, Christopher Jankowski, Jorge Verdecia, Carmen Isache, Malleswari Ravi, Yvette McCarter, Anthony M Casapao

**Affiliations:** UF Health Jacksonville, Jacksonville, Florida; UF Health Jacksonville, Jacksonville, Florida; University of Florida COM-Jacksonville, Jacksonville, Florida; UF Health Jacksonville, Jacksonville, Florida; University of Florida COM-Jacksonville, Jacksonville, Florida; UF Health Jacksonville, Jacksonville, Florida; University of Florida College of Pharmacy, Jacksonville, FL

## Abstract

**Background:**

*Staphylococcus aureus* bacteremia (SAB) is one of the leading causes of bloodstream infections complicated by metastatic diseases leading to extended lengths of stay (LOS). Infectious disease (ID) consultation is associated with greater adherence to standards of care and improved clinical outcomes. There are sparse data evaluating the impact of the timing for ID consult on SAB management. The purpose of this study was to compare the difference in clinical outcomes in patients with SAB who received an early (< 5 days) versus late (≥ 5 days) ID consult.

**Methods:**

This was an IRB-approved, retrospective, cohort study in hospitalized adult patients with SAB who received an ID consultation between 2015 and 2020. The timing of the ID consult was defined from the order of the index positive blood culture to the placement of the consult. The composite primary endpoint included: identifying the source of SAB, follow-up cultures, obtaining an echo, use of parenteral therapy, and treatment duration. Secondary endpoints included mortality, recurrence, LOS, duration of bacteremia, and readmission rates (30, 60, and 90-day). A machine learning algorithm (MLA) was utilized to determine an alternative to the 5 days threshold for early vs late.

**Results:**

A total of 321 patients with SAB were included with baseline characteristics (Table 1). The early group was more likely to meet the primary endpoint (62.5% vs. 28.3%, *p* < 0.001). The median duration of bacteremia and length of stay was shorter in the early group (*p* < 0.001) (Table 2). MLA determined an alternative threshold of ≤ 3.5 days for early ID consult. Multivariable analyses reported early ID consult in ≥ 5 days (adjusted OR: 6.42, 95% CI [3.20, 12.89]; *p* < 0.001) and ≤ 3.5 days (adjusted OR: 3.47, 95% CI [2.08, 5.81]; *p* < 0.001) as independent predictors for achieving the five quality care indicators (Table 3).

**Figure:**
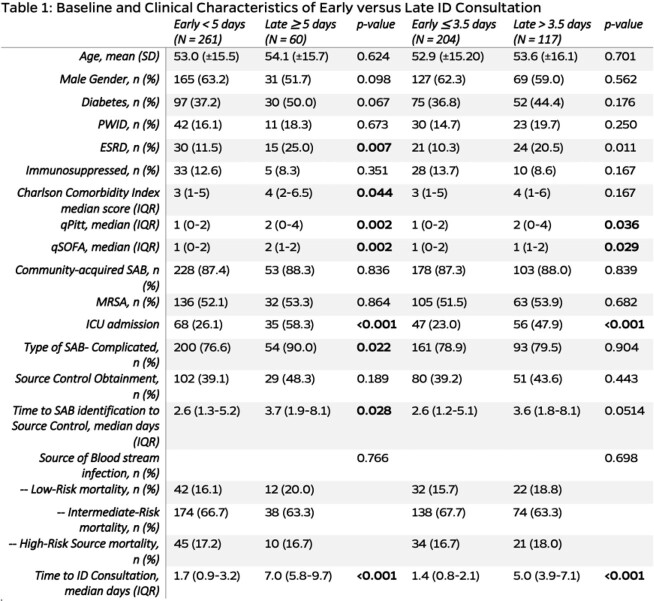

Comparing the predefined definition of early infectious diseases consultation as < 5 days and late infectious diseases consultation as >= 5 days in univariate analyses to baseline and clinical characteristics. A comparison of an alternative threshold of 3.5 days was also performed in univariate analyses. PWID: persons who inject drugs; ESRD: end-stage renal disease; SD: standard deviation; IQR: interquartile range; MRSA: methicillin-resistant Staphylococcus aureus; ICU: intensive car unit.

**Figure:**
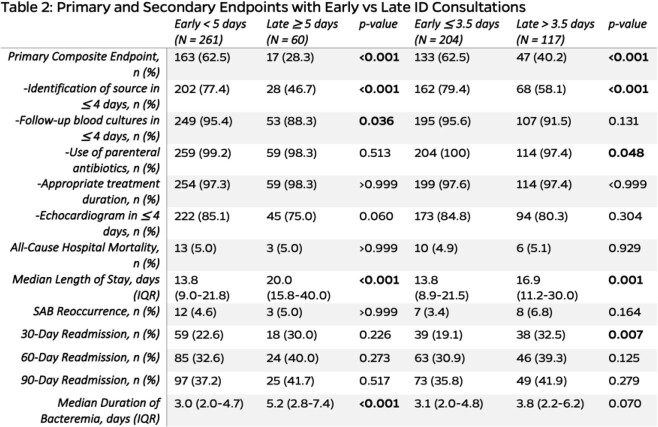

Comparing the predefined definition of early infectious diseases consultation as < 5 days and late infectious diseases consultation as >= 5 days in univariate analyses to primary and secondary endpoints. A comparison of an alternative threshold of 3.5 days was also performed in univariate analyses.

**Figure:**
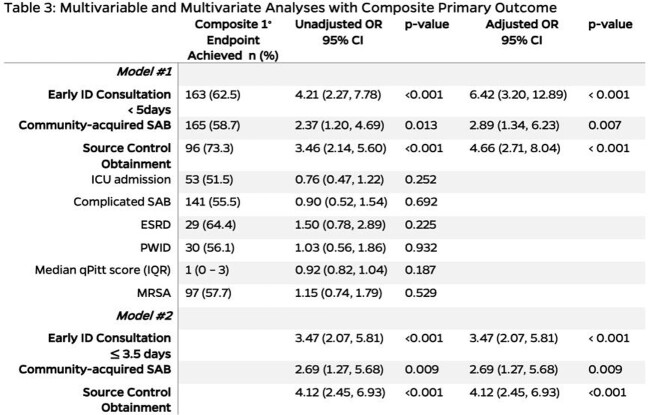

Bivariate analyses were performed with covariates of interest that may be associated with five quality indicators of Staphylococcus aureus bacteremia management (composite primary endpoint). Multivariable analysis was performed to control for covariates statistically significant in the bivariate analyses. Early infectious diseases consultation < 5 days was an independent predictor for meeting the five quality indicators of SAB management. Another multivariable analysis confirmed early ID consultation <= 3.5 days as an independent predictor for meeting the five quality indicators of SAB management.

**Conclusion:**

Patients with SAB who received an early ID consultation were more likely to achieve the quality care indicators and have a shorter duration of bacteremia and LOS. These data suggest that patients with SAB should have an ID consultation < 5 days from index culture acquisition. Future studies should evaluate if an earlier time frame of 3.5 days for ID consultation improves clinical outcomes.

**Disclosures:**

**All Authors**: No reported disclosures.

